# *Bacillus pumilus* increases boron uptake and inhibits rapeseed growth under boron supply irrespective of phosphorus fertilization

**DOI:** 10.1093/aobpla/plz036

**Published:** 2019-06-26

**Authors:** Sajid Masood, Xue Qiang Zhao, Ren Fang Shen

**Affiliations:** State Key Laboratory of Soil and Sustainable Agriculture, Institute of Soil Science, Chinese Academy of Sciences, Nanjing, China

**Keywords:** boron, phosphorus, plant growth-promoting bacteria, soil acidification

## Abstract

The present study was carried out to investigate how plant growth-promoting bacteria (PGPB) influence plant growth and uptake of boron (B) and phosphorus (P) in rapeseed (*Brassica napus*). Rapeseed was subjected to control, B, P and B + P treatments, either with or without *B. pumilus* (PGPB) inoculation, and grown in pot culture for 6 weeks. In the absence of *B. pumilus*, the addition of B, P or both elements improved the growth of rapeseed compared with the control. Interestingly, *B. pumilus* inoculation inhibited plant growth and enhanced B uptake under B and B + P but not under control and P conditions. In addition, *B. pumilus* inoculation decreased the pH of soil under B and B + P supplies. *Bacillus pumilus* inoculation thus increased rapeseed B uptake and inhibited growth under B supply, which suggests that the effects of PGPB on rapeseed growth depend on the addition of B to soil. *Bacillus pumilus* inoculation may therefore be recommended for the enhancement of rapeseed B levels in B-deficient soils but not in B-sufficient ones.

## Teaser

The present study indicates that *Bacillus pumilus* (plant growth-promoting bacteria, PGPB) enhances B uptake by rapeseed, which is consistent with previous reports. However, interestingly, increased B accumulation in rapeseed inhibited the growth of rapeseed under B-sufficient soil conditions. These inhibitory effects of *B. pumilus* on rapeseed growth were related to soil acidification, which increased the B availability in the soil–plant system. This novel information on the role of PGPB in B nutrition and plant growth improves our understanding of how PGPB influence B uptake and has potential implications for instructing the application of PGPB in agriculture.

## Introduction

Boron (B) is a micronutrient essential for plant growth and development, and rapeseed (*Brassica napus*) is a globally important oil crop with high B requirements (Xu *et al.* 2002). In China, rapeseed is grown on over 6.67 million ha, in which levels of B are either moderately or extremely insufficient for plant growth (Xu *et al.* 2002). B deficiency is a major factor limiting the growth of at least 132 crops including rapeseed in over 80 countries (Shorrocks 1997). B-deficiency symptoms, which are often visible in rapidly growing tissues, lead to growth reduction (Dell and Huang 1997). Adequate B nutrition is thus recommended to overcome B deficiency in plants. Because a narrow range exists between B deficiency and B toxicity, however, the application of exogenous B can easily be lethal to plants.

Phosphorus (P) is an essential macronutrient required by plants for normal metabolic processes, such as energy transfer, macromolecule biosynthesis, signal transduction, photosynthesis and respiration (Huang *et al.* 2008). Soil P availability is also a major problem, as this element is adsorbed onto the surface of soil particles, where it readily forms insoluble compounds after reacting with soil cations such as iron, aluminium and calcium. As a result, as much as 90 % of soil P, which is required for plant growth, may no longer be available for uptake.

Because both B and P are essential in higher plants, B–P interactions in agricultural crops have been a subject of interest (Yamanouchi 1980; Günes and Alpaslan 2000). Although many reports have appeared on their interactions in plants, the mutual effect of these two elements on plants is unclear. Some studies have found that P supply reduces B in tissues of different plant species (Salinas *et al.* 1986; Kaya *et al.* 2009) and vice versa (May and Pritts 1993; Günes and Alpaslan 2000), while others have uncovered synergistic effects of B and P on various plant metabolic processes (Chatterjee *et al.* 1990). Despite this antagonism between B and P with respect to tissue element concentrations, an inadequate supply of both nutrients greatly affects plant physiological processes, including ionic balance. In this regard, B deficiency has been shown to impair plant vegetative growth (Marschner 2011) and nutrient acquisition (Koshiba *et al.* 2009). Similar to B, an imbalance in plant P nutrition alters the nutrient uptake pattern of plants (Biddinger *et al.* 1998).

Plant growth-promoting bacteria (PGPB) and other soil microorganisms may improve plant growth by increasing water uptake and nutrient availability (Dey *et al.* 2004), including B and P availability. Furthermore, an increase in plant water content due to PGPB inoculation can contribute to ion homeostasis in plants and may improve plant mineral nutrition (Dodd and Perez-Alfocea 2012), including that of B (Khan *et al.* 2016). The enhancement of plant mineral nutrition resulting from PGPB inoculation has mainly been attributed to soil acidification caused by organic acid exudation (Israr *et al.* 2016). Likewise, much information is available on the beneficial role of PGPB strains, such as *Acinetobacter*, *Alcaligenes*, *Arthrobacter*, *Azospirillum*, *Azotobacter*, *Bacillus*, *Beijerinckia*, *Burkholderia*, *Enterobacter*, *Erwinia*, *Flavobacterium*, *Rhizobium* and *Serratia*, on plant growth (Bashan and de-Bashan 2005; Han and Lee 2005; Huang *et al.* 2017), but the mechanisms of action differ depending on the PGPB strain, soil conditions and plant species. For example, Stavropoulou (2011) reported that *Bacillus subtilis* improves tomato growth by inducing the production of metabolites. In our recent study, however, *Bacillus pumilus* inoculation caused B to accumulate in rice grown under excess B conditions, but its growth-promoting effect was due to the induced production of plant antioxidants (Khan *et al.* 2016).

Boric acid is mostly uncharged under physiological conditions, and its uptake is influenced by the transpiration stream (Alpaslan and Günes 2001; Reid 2014). Plant growth-promoting bacteria can increase B accumulation in plants through enhancing transpiration-driven water flow and may cause B toxicity. Soil pH is another relevant factor. For example, PGPB-assisted enhancement of the mineral nutrition of chickpea grown in soil at a pH of 7.5 has been found to be due to root exudation-mediated soil acidification (Israr *et al.* 2016). Previous studies have generally focused on the effects of PGPB on the growth and nutrient acquisition of plants grown in neutral to alkaline soils, with relatively little attention paid to plants in low-pH soils, where PGPB may function differently. Because rapeseed is very sensitive to both P and B deficiencies, we investigated the effects of *B. pumilus* inoculation on the growth and B and P uptake of rapeseed grown in low-pH soil. Our findings, which should aid understanding of how B, P and PGPB interact, have potential implications for the enhancement of P and B nutrition in rapeseed.

## Materials and Methods

### Experimentation and harvesting

A pot experiment with rapeseed cultivar Westar 10 (Xu *et al.* 2002) was conducted under greenhouse conditions in Nanjing, China (32.09°N, 118.83°E). Plants were grown under day/night temperatures of 22/16 °C, a relative humidity of 65–70 %, and 11–12 h of daily illumination from natural sunlight passing through the glass. The soil for the present experiment was collected from a rice–rapeseed rotation field (Hengxi, Nanjing). Surface soil samples (0–15 cm depth) were collected, air-dried, sieved (<2 mm) and mixed completely to ensure uniformity. Afterwards, the soil was autoclaved at 121 °C for 20 min (Jorquera *et al.* 2013; Sirajuddin *et al.* 2016), and the following basic properties were determined: EC, 300 µS cm^−1^; pH, 5.58; organic matter, 23.6 g kg^−1^; available N, 202 mg kg^−1^; available P, 11.04 mg kg^−1^; extractable K, 58 mg kg^−1^; extractable Ca, 818 mg kg^−1^; extractable Mg, 155 mg kg^−1^; and available B, 0.40 mg kg^−1^. Each pot (15 cm height and 14.5 cm diameter) contained 1.5 kg of sterilized soil. Four fertilizer treatments were applied: control (no B or P), B (2 mg B kg^−1^ soil), P (100 mg P kg^−1^ soil) and B + P (2 mg B kg^−1^ soil + 100 mg P kg^−1^ soil). B and P were applied as H_3_BO_3_ and Ca(H_2_PO_4_)_2_·H_2_O, respectively. In general, a B soil concentration lower than 0.5 mg kg^−1^ is considered insufficient for plants such as dicots, whereas a level higher than 1 mg B kg^−1^ soil is thought to be adequate. Accordingly, B was added at a concentration of 2 mg kg^−1^ soil. N and K were applied in all treatments at rates of 150 mg N kg^−1^ soil and 100 mg K kg^−1^ soil in the form of urea and KCl, respectively.

After surface sterilization with 10 % H_2_O_2_ for 20 min, seeds were sown in the soil-filled pots. After 4–7 days of seed germination, redundant seedlings were eliminated, and five uniform seedlings were maintained in each pot. On Day 11, the soil in a subset of the experimental pots was inoculated with a culture of *B. pumilus* obtained from the Agricultural Culture Collection of China (ACCC 19290). To ensure a uniform cell density (10^8^ CFU mL^−1^), the bacterial suspension was diluted and maintained at an optical density of 1.0 at 535 nm using a spectrophotometer. The bacterial suspension was subsequently applied by surface irrigation (10^8^ cells per pot) as well as by injection into the soil around each *Brassica* seedling using a micropipette (10^7^ cells per seedling). For this purpose, fresh bacterial culture was prepared in 100 mL Lysogeny Broth (LB) medium, incubated overnight at 30 ± 2 °C in a shaker-incubator (THZ-98C, Shanghai Bluepard Instruments, Shanghai, China) and centrifuged at 1610 *g* for 20 min. The resulting pellet was then diluted with sterile water and used for seedling inoculation. Four replications per treatment were performed, both with and without *B. pumilus* inoculation. Soils were irrigated with deionized water throughout the experiment. Plants were harvested 6 weeks after *B. pumilus* inoculation, oven-dried at 65 °C and subsequently used for shoot mineral analysis. Similarly, soil samples from each pot were collected after plant harvest, air-dried, sieved (<2 mm) and analysed for soil pH and mineral concentrations.

### Leaf transpiration and photosynthesis measurements

Leaf transpiration and photosynthetic rates of intact leaves (2–3 middle leaves) were measured before harvest with a portable photosynthesis apparatus (LI-COR 6400, Lincoln, NE, USA). Measurements were performed under a constant air flow, with the light intensity first adjusted to 1500 μmol m^−2^ s^−1^.

### Analyses of shoot B and P

Dried, ground shoot samples (100 mg) were digested with a mixture of HNO_3_/HClO_4_ (4:1 [v/v]) in digestion blocks until clear digests were obtained. The shoot digests were then diluted with 20 mL of Millipore water and used for determination of B and P on an inductively coupled plasma–optical emission spectrometer (ICP-OES) (iCAP 7000 Series, Thermo Fischer Scientific, Bremen, Germany) coupled to an ASX-520 autosampler.

### Analyses of soil B and P

After extraction with boiling water for 10 min according to the method of Berger and Truog (1939), available B in soil samples was quantified by ICP-OES. Similarly, available P was measured by ICP-OES following extraction from soil samples using NaHCO_3_ solution according to the protocol of Olsen *et al.* (1954).

### Determination of soil bacterial populations at plant harvest

The total bacterial population of soils after plant harvest was determined according to the protocols of Travers *et al.* (1987) and Molina *et al.* (2010) with slight modifications. Briefly, 1 g of fresh soil and 10 mL sterile water were added to a 250-mL Erlenmeyer flask and mixed with shaking at 200 rpm for 5 h. Afterwards, 1 mL of bacterial suspension was added to a 1.5-mL Eppendorf tube and heat-shocked at 80 °C for 5 min to eliminate non-sporulated microbes. Finally, 100 μL of a serial dilution of the suspension was spread on LB medium containing 25 μg mL^−1^ of the antibiotics purine, streptomycin and putrescence dihydrochloride, and bacterial colonies were counted.

### Experimental design and data analysis

The entire experiment was repeated twice, during two different years, i.e. 2015 and 2016 (October–November), and consistent results were obtained. The experiment was set up using a completely randomized design with factorial arrangements. All data were subjected to analysis of variance, and treatment means were compared by Tukey’s test (*P* ≤ 0.05) in SigmaStat (SPSS, Inc., Chicago, IL, USA).

## Results

### Rapeseed growth and biomass

**Figure 1.**
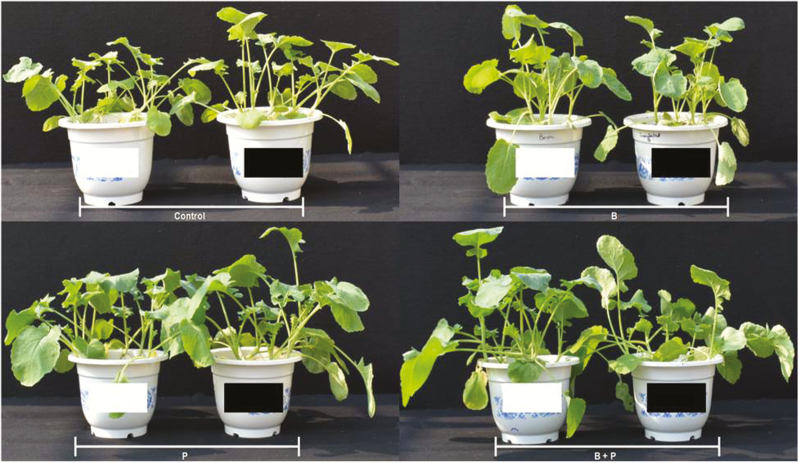
Growth response of rapeseed to boron (B) and phosphorus (P) supplies, either with (black) or without (white) *B. pumilus* inoculation.

**Figure 2.**
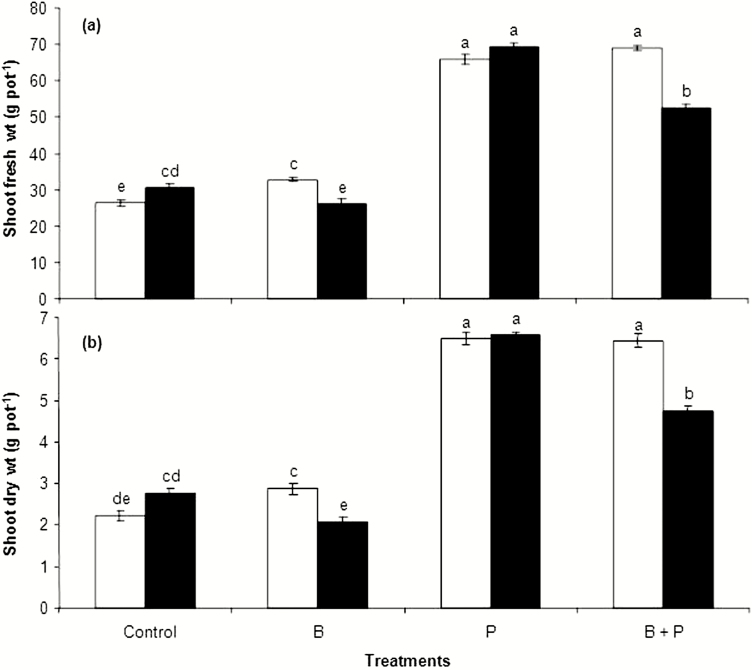
The effects of boron (B) and phosphorus (P), either with (black) or without (white) *B. pumilus* inoculation, on shoot fresh (A) and dry (B) weights of rapeseed. Values on the *y*-axis are means ± standard error of four replicates. Different letters above columns indicate significant differences among treatments at the *P* ≤ 0.05 level.

Plants, included with *B. pumilus* in presence of B applications (either solely or in combination with P), seemed to be chlorotic and stunted with upwardly cupped leaves and in some cases the leaf edges were burnt (Fig. 1). These symptoms were not observed on the other treatments. For non-inoculated plants, the three treatments, i.e. B, P and B + P, increased significantly rapeseed shoot fresh and dry weights when compared with the non-amended control treatment (Fig. 2A and B). For inoculated plants, P and B + P, but not B, increased shoot fresh and dry weights when compared with the non-amended control treatment (Fig. 2A and B). Inoculation with *B. pumilus* slightly enhanced shoot fresh and dry weights of rapeseed under the control conditions, but not under P treatment when compared with the non-inoculation (Fig. 2A and B). On the other hand, *B. pumilus* inoculation decreased the fresh and dry weights of plant shoots under B and B + P treatments. The increases that occurred in shoot fresh and dry weights of inoculated plants amended with B + P were significantly lower than those obtained for the sole application of P. These results probably highlight the negative impacts of B applications on the growth of rapeseed inoculated with *B. pumilus*. Although, we tried to separate roots from the soil by various methods; however, most roots were intermixed with soil particles and clods, as rapeseed has a very fine root system. Consequently, root fresh and dry weights were not recorded.

### Transpiration and photosynthesis in intact leaves

**Figure 3.**
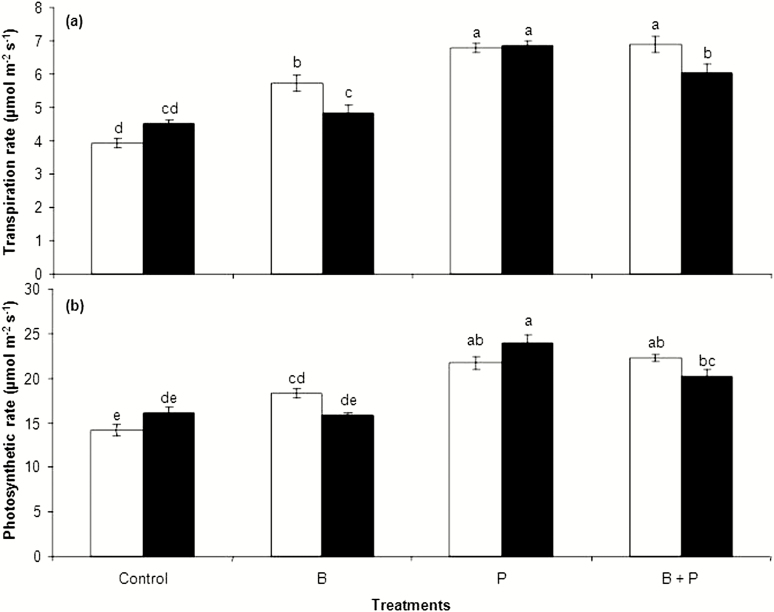
The effects of boron (B) and phosphorus (P), either with (black) or without (white) *B. pumilus* inoculation, on leaf transpiration (A) and photosynthetic rate (B) of rapeseed. Values on the *y*-axis are means ± standard error of four replicates. Different letters above columns indicate significant differences among treatments at the *P* ≤ 0.05 level.

Similar to their effects on plant biomass, non-inoculated B, P and B + P treatments increased significantly leaf transpiration and photosynthetic rates when compared with the control treatment (Fig. 3A and B). Generally, the highest transpiration and photosynthetic rates were recorded for plants amended with either P solely or in combination with B. It seems that *B. pumilus* inoculation had no significant effect on leaf transpiration and photosynthetic rates of rapeseed subjected to P treatment or even the control one (Fig. 3A and B); however, inoculation with *B. pumilus* decreased significantly leaf transpiration rate with no significant effect on plant photosynthesis under B and B + P treatments (Fig. 3A and B).

### Concentrations of B and P within plants and the corresponding available concentrations in soil after plant harvest

**Figure 4.**
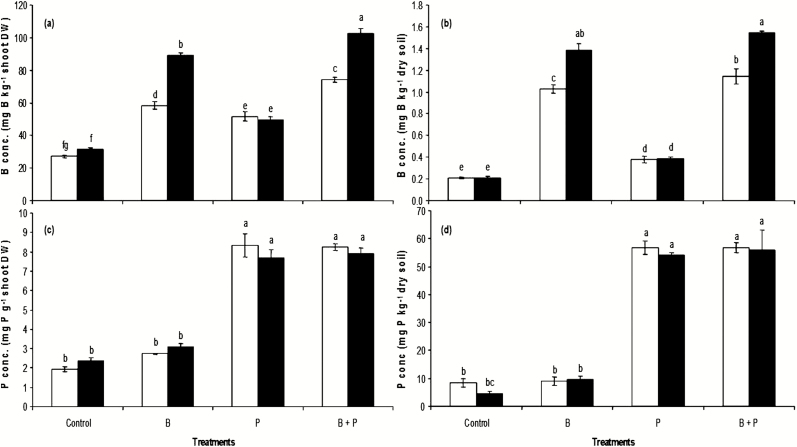
The effects of boron (B) and phosphorus (P), either with (black) or without (white) *B. pumilus* inoculation, on shoot B (A), soil B (B), shoot P (C) and soil P (D). All values are means ± standard error of four replicates. Different letters above columns indicate significant differences among treatments at the *P* ≤ 0.05 level.

Regardless of whether inoculation with *B. pumilus* was performed or not, the three treatments, namely, B, P and B + P, increased shoot and soil available concentrations of B compared with the control. In this concern, the combined application of B and P resulted in the highest concentrations of B within plant parts and soil (Fig. 4A and B). Inoculation with *B. pumilus* enhanced rapeseed shoot and soil B concentrations under B and B + P treatments but had no significant effect on these parameters in the control and P-treated plants (Fig. 4A and B).

On the other hand, the concentrations of P increased significantly within plants in presence or absence of *B. pumilus* inoculation for ‘P’ and ‘B + P’ treatments but not for ‘B’ treatment. Concurrent increases in soil available P occurred also in soils for the above-mentioned treatments. It seems that *B. pumilus* inoculation had no further significant effects on shoot and soil P concentrations regardless of treatment (Fig. 4C and D). Overall, these results suggest that P fertilization together with plant inoculation with *B. pumilus* enhances B uptake by rapeseed; however, B fertilizer and *B. pumilus* do not affect P uptake. In addition, *B. pumilus* did not solubilize P on Pikovskaya medium, as no clear halozones were observed **[see****Supporting Information—Fig. S1****]**, further suggesting that *B. pumilus* is not effective for increasing P bioavailability.

### Soil pH after plant harvest

**Figure 5.**
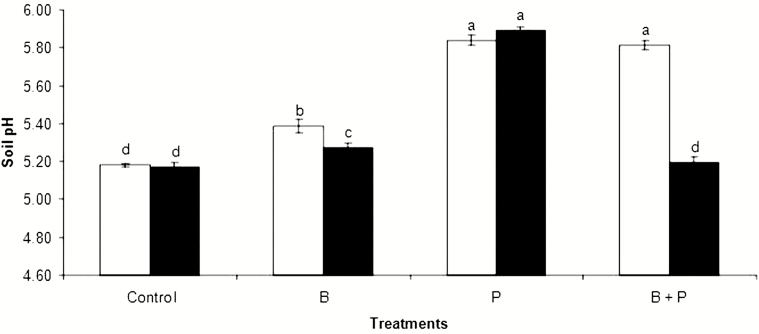
The effects of boron (B) and phosphorus (P), either with (black) or without (white) *B. pumilus* inoculation, on soil pH. All values are means ± standard error of four replicates. Different letters above columns indicate significant differences among treatments at the *P* ≤ 0.05 level.

Without *B. pumilus* inoculation, B, P and B + P treatments all increased the soil pH compared with the control (Fig. 5). With *B. pumilus* inoculation, P treatment resulted in higher soil pH values than that observed ones upon control, B or B + P treatments (Fig. 5). In addition, *B. pumilus* inoculation significantly lowered the soil pH especially when combined with B and B + P treatments but not the control and P treatments (Fig. 5). Accordingly, it can be deduced that *B. pumilus* has acidifying effects on B-sufficient soils.

### Soil bacterial population after plant harvest

**Figure 6.**
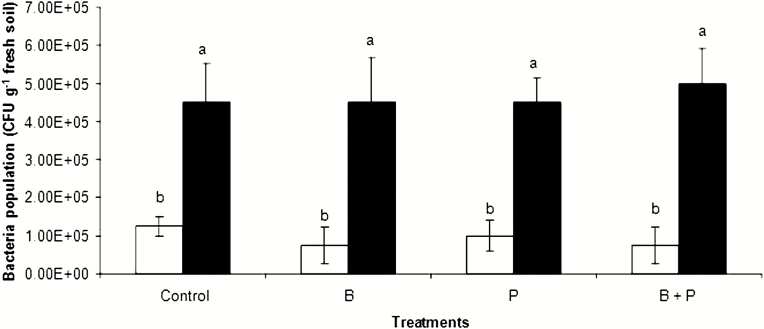
The effects of boron (B) and phosphorus (P), either with (black) or without (white) *B. pumilus* inoculation, on the soil bacterial population after plant harvest. All values are means ± standard error of four replicates. Different letters above columns indicate significant differences among treatments at the *P* ≤ 0.05 level.

Without *B. pumilus* inoculation, none of the treatments influenced the bacterial population, which had background levels of ~1 × 10^5^ CFU g^−1^ fresh soil (Fig. 6). On the other hand, *B. pumilus* inoculation enhanced the bacterial population by 4- to 5-fold under all treatments, suggesting the survival of the *B. pumilus* strain used in our experiment.

## Discussion

### *Bacillus pumilus* inoculation inhibited rapeseed growth while increased B uptake under B addition

In the present study, *B. pumilus* inoculation inhibited rapeseed growth while enhanced plant B uptake under B and B + P supplies but not under control conditions or P supply. Although B is essential for plant growth, high B concentrations can be toxic to plants. In barley, high B concentrations (62.4 mg B kg^−1^) have been found to affect plant growth and reduce grain yield by 17 % (Cartwright *et al.* 1984). In some plants, the B toxicity threshold level is as low as a few mg kg^−1^ (Hillel 2000). In the current study, shoot B concentrations exceeded sufficiency limits (5–25 mg B kg^−1^; Bergmann 1992). Numerous researchers have also reported that B toxicity in crops may occur in the range of 10–130 mg B kg^−1^ dry weight (Riley *et al.* 1994; Alpaslan and Günes 2001; Mengel and Kirkby 2001). According to our results, enhanced B accumulation in rapeseed reduced photosynthetic and transpiration rates, finally leading to chlorosis and burning of leaf margins. These observations are in line with the findings of Landi *et al.* (2013), who found that excess B application decreases the leaf transpiration and photosynthetic rates of cucumber and zucchini. Similarly, excess B also decreases the transpiration rates of tomato (Ben-Gal and Shani 2002) and date palm (Tripler *et al.* 2007). Once B enrichment takes place in plant tissues, this process affects certain plant physiological processes, such as cell division (Liu and Yang 2000), and leads to shoot growth reduction (Reid *et al.* 2004). Similarly, high B concentrations tend to reduce the shoot biomass of crops such as lettuce (Eraslan *et al.* 2007), pepper (Yermiyahu *et al.* 2008), tomato (Sirajuddin *et al.* 2016), broccoli (Smith *et al.* 2013) and wheat (Masood *et al.* 2012, 2016). In addition, the visual leaf symptoms observed in rapeseed in our study are congruent with the leaf B toxicity symptoms reported by Smith *et al.* (2013). We thus hypothesize that the inhibitory effects of *B. pumilus* on rapeseed growth are related to increased B uptake under B-sufficient conditions.

**Figure 7.**
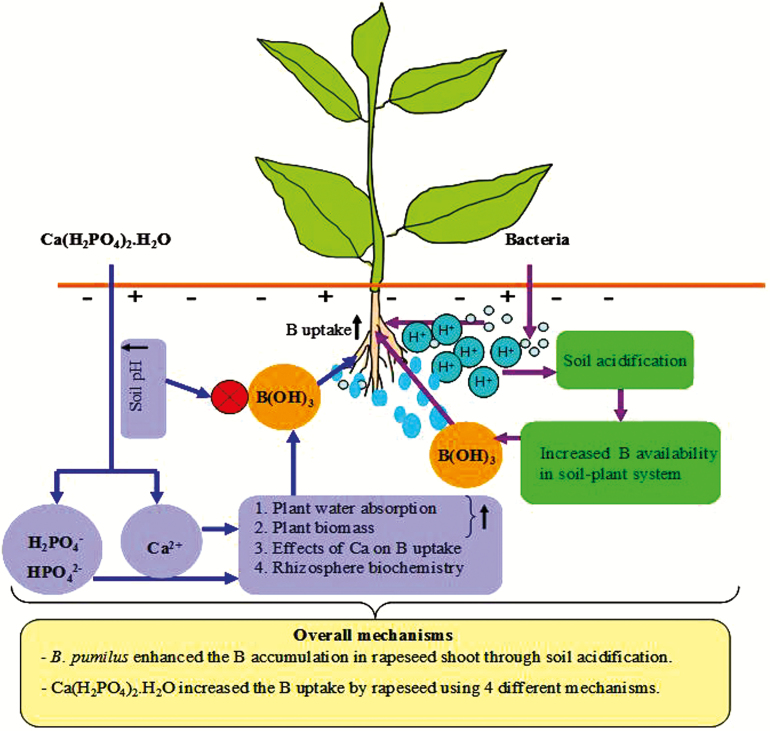
Schematic diagram of mechanisms responsible for enhanced B uptake by plants subjected to both *B. pumilus* (bacterial) inoculation and P addition. The upper right part represents mechanisms underlying increased B availability in the soil–plant system under *B. pumilus* inoculation, whereas the upper left part corresponds to mechanisms increasing B availability in the soil–plant system under P supply. The bottom box summarizes the overall mechanisms contributing to higher B accumulation in rapeseed grown under normal B and P supplies.

*Bacillus pumilus* inoculation may enhance plant B uptake by increasing B availability in the soil–plant system through soil acidification (Fig. 7). The responsible mechanism is PGPB-induced production of organic acids into the soil (Deubel *et al.* 2000), which in turn may decrease soil pH and increase B availability to plants. Increasing evidence is appearing that PGPB inoculation decreases soil pH through the production of organic acids as secondary metabolites (Turan *et al.* 2006). Similar results have been obtained by Orhan *et al.* (2006), who observed that PGPB inoculation tends to decrease soil pH and stimulate nutrient availability. The soil acidification may be caused by acid produced by *B. pumilus* itself and/or secreted by plant roots in the presence of *B. pumilus*. According to our results, *B. pumilus* inoculation decreased the soil pH only under B and B + P conditions, not under control conditions or P supply. Inoculation with the strain, however, significantly increased the soil bacterial population under all treatments.

In the present study, *B. pumilus* inoculation also decreased the soil pH while increased B availability in the soil–plant system under B-sufficient conditions. In a previous investigation, we also recorded higher B concentrations in rice following *B. pumilus* inoculation (Khan *et al.* 2016). Total B contents in soil may vary from 2 to 200 mg kg^−1^, little of which (5–10 %) is available to plants (Diana 2006). This situation is due to the involvement of chemical processes that affect sorption reactions and limit B availability to plants by controlling dissolved B concentrations (Ranjbar and Jalali 2014). Plants can only use dissolved B, and a large fraction of B is adsorbed onto the soil surface, where it is non-toxic to plants (Keren *et al.* 1985). Under such conditions, PGPB may enhance B availability in the soil–plant system through acidification. According to Goldberg *et al.* (2008), B adsorption onto the soil surface increases as the pH increased from 3 to 9. This result implies that a lower pH favours desorption of B from the soil surface, thereby enhancing B mobility in soil and consequently increasing plant B uptake. Soil acidification is therefore probably the mechanism responsible for the enhanced uptake of B by rapeseed inoculated with *B. pumilus*.

Plant water absorption can also have certain positive or negative effects on plant B uptake. Because B uptake is greatly influenced by the transpiration stream (Alpaslan and Günes 2001), an increase in plant water absorption under PGPB supply (Mayak *et al.* 2004; Dodd and Perez-Alfocea 2012) may enhance B uptake. On the one hand, the transpiration of rapeseed in the present study was decreased by *B. pumilus* inoculation under B-sufficient conditions; thus, it can be deduced that increased plant water absorption is not associated with increased B uptake. On the other hand, published studies are available in which PGPB enhanced nutrient uptake by plants through promoting root growth (Yildirim *et al.* 2011). As mentioned in the Results section, we could not collect the whole roots of rapeseed; consequently, we could not determine whether or not root growth promotion is responsible for *B. pumilus*-induced B toxicity.

In a previous study, *B. pumilus* was found to enhance the B tolerance of tomato plants grown in alkaline soil (pH 7.7) by inducing the activity of the antioxidant enzymes superoxide dismutase and catalase (Sirajuddin *et al.* 2016). This result is not consistent with our present findings that *B. pumilus* increased B uptake and inhibited rapeseed growth under B supply in acidic soil (pH 5.58), which suggests that antioxidant enzymes did not function effectively under our experimental conditions. The effects of *B. pumilus* on B uptake and tolerance may therefore depend on soil pH and plant species.

### *Bacillus pumilus* inoculation did not influence P uptake by rapeseed

Although some investigations have indicated that PGPB can enhance P uptake by plants (Dey *et al.* 2004; Israr *et al.* 2016), *B. pumilus* inoculation had no effects on rapeseed P uptake in our study, and *B. pumilus* exhibited no P-solubilization potential against insoluble P. In addition, the soil used for our experiments was acidic (pH 5.58), which probably diminished the effects of *B. pumilus* on rapeseed P uptake even though *B. pumilus* caused soil acidification. These results suggest that the P-solubilization function varies among different PGPB strains, soil types and plant species.

### P enhanced B uptake, but B did not enhance P uptake by rapeseed

Our results reveal that P addition enhanced rapeseed B uptake and available soil B concentrations. Such enhancement may have occurred through one of the following mechanisms (Fig. 7): (i) increased plant water uptake; (ii) improved plant growth; (iii) calcium effects on B availability in the soil–plant system or (iv) increased soil B availability resulting from plant-induced modifications in rhizosphere biochemistry. P is well known to enhance plant water uptake and water use efficiency (Suriyagoda *et al.* 2014). In the present study, an increase in B uptake induced by addition of P was related to enhanced water uptake and increased plant biomass, as P produced higher shoot weights and enhanced leaf transpiration. As B is transported mainly through the transpiration stream, an increase in plant water absorption may enhance rapeseed B uptake. Our results are in agreement with the findings of Gupta (1993) and Kizilgoz and Sakin (2015), who reported significant increases in B accumulation in plants at optimum P levels. By contrast, antagonistic interactions between B and P uptake have been observed in maize (Günes and Alpaslan 2000) and tomato (Kaya *et al.* 2009). To explain the results of their study, Günes and Alpaslan (2000) proposed that phosphate and borate anions are transported by the same physiological mechanisms, whereby a competition might exist between these two anions. The inconsistency among these different study results may be due to differences in soil properties, plant types, fertilizer sources and application methods. Because P treatment increased the soil pH, the increased B uptake by rapeseed may be unrelated to pH and may instead be due to the addition of a calcium source for P. In general, calcium can increase B uptake by plants, especially under B-deficient conditions (Carpena *et al.* 2000). Increased B mobilization in crops as a result of calcium supply to the root medium has also been suggested by Tisdale *et al.* (1985). This suggestion is based on the idea that both B and calcium may influence their availability in plants (Teasdale and Richards 1990), as both elements have structural roles in plant cell walls. Overall, the positive effects of increased plant water uptake, improved plant growth and calcium on rapeseed B uptake exceeded the negative effects of high soil pH.

Although P enhanced rapeseed B uptake in the present study, B did not influence P uptake. Neither shoot nor soil P was enhanced by B treatment, which suggests that B has no effects on available soil and shoot P. The fact that soil and shoot P are independent of B may be due to the uncharged nature of B. Similar results, i.e. high B concentrations do not significantly correlate with P uptake, have been reported in lettuce (Petridis *et al.* 2013), maize (Aref 2012) and yellow passion fruit seedlings (da Silva Matos *et al.* 2015).

## Conclusions

Both *B. pumilus* inoculation and P addition enhanced the uptake of B by rapeseed, while inoculation inhibited the growth of rapeseed under B supply. Increased concentration of B by rapeseed subjected to *B. pumilus* inoculation was related to soil acidification, which enhanced B availability in the soil–plant system. Increased uptake of B by rapeseed under P addition was mainly attributed to increased plant water uptake, improved plant growth and the effects of calcium on B availability in the soil–plant system. Hence, *B. pumilus* inoculation of rapeseed to replenish B nutrition is only recommended under low B conditions.

## Supporting Information

The following additional information is available in the online version of this article—

**Figure S1.** Visual evidence that *Bacillus pumilus* has no effect on phosphorus (P) solubilization, namely, no clear zones were formed on Pikovskaya medium streaked with bacterial suspension.

All the original data in Figs 1–6.

plz036_suppl_Supplementary_Data

plz036_suppl_Supplementary_Figure_S1

## Sources of Funding

This work was supported by the Strategic Priority Research Program of the Chinese Academy of Sciences (nos. XDB15030302 and XDB15030202), the National Key Basic Research Program of China (no. 2014CB441000) and Chinese Academy of Sciences President’s International Fellowship Initiative (no. 2015PB054).

## Contributions by the Authors

X.Q.Z., S.M. and R.F.S. conceived the experiments. S.M. conducted the experiments, whereas S.M. and X.Q.Z. analyzed the results. S.M. wrote the main manuscript, whereas X.Q.Z. revised it critically for intellectual content. All authors reviewed and approved the manuscript.

## Conflict of Interest

None declared.
