# Supplementary material for: Bacillus pumilus increases boron uptake and inhibits rapeseed growth under boron supply irrespective of phosphorus fertilization
Source: AoB Plants. 2019 Jun 26;11(4):plz036. doi: 10.1093/aobpla/plz036 (PMC6626985; doi:10.1093/aobpla/plz036)
Supplement: plz036_suppl_Supplementary_Figure_S1 [file plz036_suppl_supplementary_figure_s1.doc]

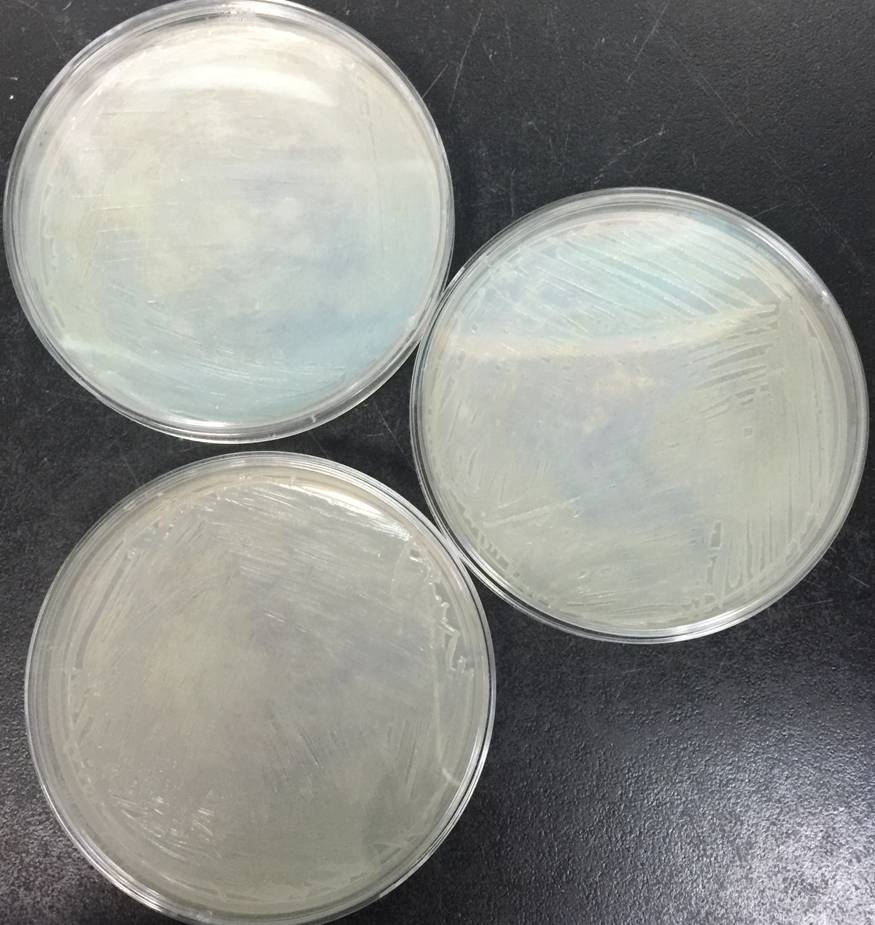


**Figure S1** Visual evidence that *B. pumilus* has no effect on phosphorus (P) solubilisation, namely, no clear zones were formed on Pikovskaya medium streaked with bacterial suspension.
